# Reducing violence and increasing condom use in the intimate partnerships of female sex workers: study protocol for *Samvedana Plus,* a cluster randomised controlled trial in Karnataka state, south India

**DOI:** 10.1186/s12889-016-3356-7

**Published:** 2016-07-29

**Authors:** Tara S. Beattie, Shajy Isac, Parinita Bhattacharjee, Prakash Javalkar, Calum Davey, T. Raghavendra, Sapna Nair, Satyanarayana Ramanaik, D. L. Kavitha, James F. Blanchard, Charlotte Watts, Martine Collumbien, Stephen Moses, Lori Heise

**Affiliations:** 1 Department of Global Health and Development, London School of Hygiene and Tropical Medicine, 15-17 Tavistock Place, London, WC1H 9SN UK; 2 Karnataka Health Promotion Trust, Bangalore, India; 3 University of Manitoba, Winnipeg, Canada

**Keywords:** Female sex work, India, Violence, HIV, STI, Intimate partner violence, Domestic violence, Condom use, Cluster Randomised Controlled Trial (RCT)

## Abstract

**Background:**

Female sex workers (FSWs) are at increased risk of HIV and STIs compared to women in the general population, and frequently experience violence in their working and domestic lives from a variety of perpetrators, which can enhance this risk. While progress has been made in addressing violence by police and clients, little work has been done to understand and prevent violence by intimate partners (IPs) among FSW populations.

**Methods:**

Samvedana Plus is a multi-level intervention programme that works with FSWs, their IPs, the sex worker community, and the general population, and aims to reduce violence and increase consistent condom use within these ‘intimate’ relationships. The programme involves shifting norms around the acceptability of beating as a form of discipline, challenging gender roles that give men authority over women, and working with men and women to encourage new relationship models based on gender equity and respect. The programme will aim to cover 800 FSWs and their IPs living in 47 villages in Bagalkot district, northern Karnataka. The study is designed to assess two primary outcomes: the proportion of FSWs who report: (i) physical or sexual partner violence; and (ii) consistent condom use in their intimate relationship, within the past 6 months. The evaluation will employ a cluster-randomised controlled trial design, with 50 % of the village clusters (*n* = 24) randomly selected to receive the intervention for the first 24 months and the remaining 50 % (*n* = 23) receiving the intervention thereafter. Statisticians will be blinded to treatment arm allocation. The evaluation will use an adjusted, cluster-level intention to treat analysis, comparing outcomes in intervention and control villages at midline (12 months) and endline (24 months). The evaluation design will involve quantitative and qualitative assessments with (i) all FSWs who report an IP (ii) IPs; and process/ implementation monitoring. Baseline data collection was completed in April 2015, and endline data collection is anticipated in May 2017.

**Conclusions:**

This is an innovative intervention programme that aims to address violence by IPs as part of HIV prevention programming with FSWs. Reducing violence is expected to reduce vulnerability to HIV acquisition, and help women to work and live without fear of violence.

**Trial registration:**

Clinical Trials NCT02807259 Jun 24 2016 (retrospectively registered).

**Electronic supplementary material:**

The online version of this article (doi:10.1186/s12889-016-3356-7) contains supplementary material, which is available to authorized users.

## Background

Female sex workers (FSWs) have a disproportionately high HIV burden compared to women in the general population [1]. In 110 countries with available data, the prevalence of HIV infection is almost 12 times higher among sex workers than for the population as a whole, with prevalence at least 50-fold higher in four countries [2]. This heightened risk of HIV acquisition and transmission among FSWs varies by sex work typology [3–6] and the nature of the underlying HIV epidemic [1]. Increased risk is due to a variety of biological (e.g. concurrent STI infection), behavioural (e.g. partner volume, partner concurrency, injecting drug use), biomedical (e.g. hormonal contraception) and structural factors (e.g. structural organisation of sex work, poverty, discrimination, housing and financial insecurity, legal and criminalisation policies, and violence) [7–10].

A growing body of evidence suggests that violence exposure is strongly associated with HIV infection, both among women in the general population [11–19], as well as among female sex workers [20–24]. However, the mechanisms through which violence operates to enhance vulnerability are multiple and complex, with HIV transmission risk occurring directly during coerced sex, as well as indirectly: FSWs who experience violence are less likely to visit STI/HIV clinics or use condoms, and more likely to experience condom breakage, report anal sex and have a concurrent STI infection, compared to FSWs who don’t experience violence [25–35]. They are also more likely to report mental health morbidity, including alcohol and drug use, which in turn can reduce their ability or will to negotiate condoms with their sexual partners [36–41]. In many settings, sex work is either illegal or the legal status is ambiguous, meaning violence by police, clients, intimate partners (IPs) and others is likely to go unreported, and the perpetrators can continue unchecked [5, 8, 42–45]. Men who are violent are more likely to report a clustering of ‘risk’ behaviours, including multiple concurrent partnerships, low/no condom use, anal sex, and substance use. These behaviours place the men and their sexual partners at increased risk of STI and HIV [14, 46–52]. In the context of female sex work, women can experience violence from a variety of perpetrators, both in their working lives (from the police, clients, pimps and street criminals) [3, 25, 45, 53–55], as well as in their ‘domestic’ lives from their non-paying, IPs (husband/lover) [35, 39, 56].

In some settings, domestic violence can be as important as ‘work-place’ violence in enhancing FSWs vulnerability to HIV and diminishing the quality of their lives [36, 57]. Domestic violence can inhibit women from negotiating condom use [15, 39, 40]. Within FSWs’ intimate partnerships, women’s ability to negotiate condom use is often severely compromised due to norms around female obedience and fidelity, men’s ignorance of her sex work status, men’s expectations that their partner will abandon sex work and are therefore no longer a source of risk, and women’s emotional and sometimes financial dependence on their partners [57–59]. All of these challenges are exacerbated if a male partner is violent.

In terms of addressing violence, among the general population, there have been several examples of successful interventions in low and middle income countries, which have worked at the community level to successfully reduce violence within intimate partnerships [60–64]. Among FSW populations, evidence suggests that comprehensive HIV prevention programming, which includes community mobilisation, empowerment and violence prevention components, can successfully reduce violence by ‘non-partners’ such as clients and the police [24, 29, 65]. However, few programmes have attempted to address violence perpetrated against FSWs by their IPs. In this paper we present the protocol for a cluster randomised control trial in northern Karnataka state in South India, to evaluate a multi-level intervention called *Samvedana Plus* which works with FSWs, IPs, sex worker community-based organisations (CBOs), and the wider community (general population) where FSWs and their IPs live, to reduce violence and increase consistent condom use within the intimate relationships of FSWs.

## Methods/Design

### The Samvedana Plus study setting

The intervention programme is located in Bagalkot district of Karnataka State, south India. This district has one of the highest HIV prevalence rates among FSWs (17.6 %) (Unpublished data, NACO High Risk Group surveillance, 2012) and among the population at large (3.5 %) (Unpublished data, NACO Antenatal surveillance, 2010) in Karnataka. It also has high rates of poverty, unemployment, illiteracy and migration. Structural and cultural factors, such as caste discrimination, poverty, and gender inequality, perpetuate the traditions of underage marriage and the dedication of young girls into sex work as part of a religious tradition, known as the *devadasi* system. The *devadasi* tradition involves a ritual in which adolescent girls are dedicated to gods and goddesses and subsequently inducted into sex work, and is the most common form of traditional sex work in north Karnataka [66]. Ninety percent of FSWs in the district are from scheduled castes or scheduled tribes – perceived as the ‘lowest’ designations in the caste system – and come from *devadasi* families. Sex work in the district is largely home-based, with girls entering sex work from 15 years of age [66]. Most communities in Karnataka accept violence against women, especially in ‘domestic’ relationships. Many people feel that violence within domestic relationships are private matters and not the responsibility of law enforcement agencies. Although there have been some initiatives from some women’s agencies to address issues of domestic violence and general violence against women, there are few efforts addressing the issue among FSW populations. In addition, there is little understanding among the general population on the rights and laws that pertain to domestic violence against FSWs.

HIV prevention programming among FSWs was initiated at scale by the government of India in 2004 across Karnataka state, and included community mobilisation, FSW empowerment and violence prevention components [67]. Violence prevention efforts began in 2006 and targeted violence perpetrated in the work place (by strangers, police, clients and street criminals), with subsequent reductions in violence reported across the state [24, 29]. However, domestic violence experienced by FSWs has not previously been addressed and most sex worker-run community based organisations (CBOs) do not feel equipped to address domestic violence. The interventions in Bagalkot are led by a sex worker collective called Chaitanya AIDS Tadegatwa Mahila Sangha (CATMS), with support from the Karnataka Health Promotion Trust (KHPT).

In northern Karnataka, initial enumeration data from FSW populations in Bagalkot and Bijapur districts (2012) suggest that most (96 %) FSWs have an IP (unpublished FSW enumeration data, KHPT, 2012). These relationships endure and evolve over time, with IPs playing physical, emotional and protective roles in the lives of women. Enumeration data suggest that 50 % of women have known their IP for more than 5 years, and that 80 % of these relationships began with the IP visiting the sex worker as a client. In addition, these relationships are often characterised by low condom use and high rates of violence, with 41 % of FSWs in northern Karnataka (Bagalkot and Bijapur districts) reporting intimate partner violence (IPV) in the past 12 months (unpublished FSW enumeration data, KHPT, 2012) and only 28 % of FSWs across Karnataka reporting consistent condom use with their IP [68].

### The Samvedana Plus intervention

Samvedana Plus works with FSWs, their IPs, the sex worker CBO and the general population where FSWs and their IPs live. It aims to reduce violence and increase consistent condom use within these intimate relationships. In developing the intervention, KHPT was able to draw upon their work reducing client and police violence against sex workers as well as their past experience implementing Stepping Stones, a curriculum based programme to reduce domestic violence among the general population in Karnataka. A Theory of Change (TOC) model was developed during a two-day workshop undertaken by the programme and research team to hypothesise the pathways through which an intervention would need to operate to reduce violence and increase condom use within intimate partnerships among FSW populations. The TOC also identified strategies to pre-empt potential barriers to implementation of the intervention (Additional file 1).

The interventions were developed following a set of assumptions:

1. The provision of immediate support will help protect FSWs from future violence.
2. Reductions in domestic violence will only occur if the intervention programme works with both the victims and the perpetrators of violence, as well as other stakeholders.
3. FSWs will be better able to negotiate safe sex with their IP if they are supported to improve their negotiation and communication skills, and have greater access to female condoms.
4. Individual and collective action against violence and/or STI/HIV transmission requires a supporting enabling environment.
5. Building the capacities of CBOs and linking them with women’s organization will strengthen the support structure.

*Samvedana Plus* is composed of innovations aimed at three levels:

(i) *Interventions with FSWs and their intimate partners*
  The intervention programme will work with FSWs and their IPs both separately via group and individual sessions, as well as together in couples’ counselling sessions. The programme will employ female facilitators to engage FSWs through a variety of activities. The objectives of working with FSWs will be to: (i) build their self-worth and individual and collective efficacy; (ii) change women’s acceptance of violence and improve their skills at negotiating safer sex; (iii) inform women about protective laws and available support services; and (iv) empower them to resist violent and risky relationships. The main FSW intervention will comprise a series of 8 modular participatory reflection sessions conducted over a 6 month period (see Table 1 for details). The reflection groups will introduce an empowerment curriculum with specific sessions focused on: women’s human rights; gender roles and norms; violence against women; building solidarity between sex workers; analysing relationships; responding to HIV-related risks; improving relationship skills and couples communication; and negotiation and use of the female condom.
  The CBO has already implemented a crisis management system, where crisis management teams are trained to intervene in the case of reports of abuse by clients, the police, or street criminals. In addition to the group reflection sessions, female facilitators will work with FSWs to develop individual safety plans; to enhance the crisis management system to respond to domestic violence incidents; to provide counselling support to discuss issues related to sex and relationships; to link FSWs to free services and commodities as needed, including free male and female condoms, as well as HIV/STI testing and treatment services; and to further provide leadership training for two FSWs from each reflection group who demonstrate emergent leadership skills (Table 2).
  The intervention will also engage with the IPs of FSWs via a series of one-to-one sessions to discuss issues around violence and condom use as well as gender, equity, respect and responsibility. Male facilitators will inform these men about the law against domestic violence and help them build communication and anger management skills. In addition to the one-to-one sessions, male facilitators will work with IPs through group reflection sessions covering a 5 module programme, conducted over two days, one month apart (see Table 3 for details). They will also link IPs to free services and commodities as needed, including free condoms and HIV/STI testing and treatment services; and select men whose behaviour changes during the intervention to become local champions for violence free relationships (Table 2).
  Work with couples will be implemented through: (i) couples counselling on an as needed basis to help resolve relationship issues; and (ii) couples events held every 3 months, which will focus on building loving and responsible relationships characterised by an absence of violence and risk (Table 2).
(ii) *Interventions with community-based organizations of FSWs*
  The programme will work with CBO members, including board members, staff and crisis management teams. The key CBO intervention component will comprise a series of two two-day workshops to build capacity of the CBO to be able to challenge violence in their own lives and support the intervention to help ensure that the project outcomes are sustained. In addition, the intervention programme will train the CBO crisis management teams to understand the context of IPV, gender equity and norms that influence IPV, and the laws that protect FSWs and other women from IPV. The programme will also support the CBO to build alliances with other networks in the district and state to help ensure that issues of violence against FSWs are integrated into the broader violence against women movement (Table 2).
(iii) *Interventions at the community level*
  At the community level, the programme will work with village and community leaders, neighbours, Panchayat Raj Institutions (local councils) and self-help groups. The main objectives of the community level interventions will be to raise awareness around IPV, domestic violence and the law, and to challenge social norms around these issues. The community level intervention will comprise: (i) folk shows and street plays that focus on issues of domestic violence and raise awareness about rights and the law; and (ii) community rallies and campaigns to raise awareness on issues of domestic violence among the general population in Bagalkot (Table 2).

**Table 1:** Workshop curriculum for female sex workers

The *first module*, will be devoted to building trust and meaningful communication, and aims to help participants appreciate the value of team support, trust, and cooperation; and understand the importance of listening to and communicating their feelings with others.
The *second module* will explore participants’ perceptions on what it means to be an ideal man and an ideal woman, examine what they feel about themselves, encourage them to be less judgmental about others, and build their self-esteem.
In the *third module* will focus on understanding relationships. Participants will describe and discuss their intimate relationships, explain their understanding and expectations of loving relationships, identify behaviours that are controlling or abusive between lovers, and consider explanations for such behaviour.
The *fourth module* will teach participants about their body, HIV-related risks in intimate relationships, and how to reduce such risks.
The *fifth module*, will focus on intimate partner violence and will help participants recognize violence in intimate relationships, categorize types of violence, consider why women tolerate violence, understand the effects of violence on women and their family and community, and refuse to tolerate violence.
The *sixth module* will teach participants about their rights and laws relating to domestic violence and violence against women, explore why women remain with violent partners, and asks women whether life without violence is possible. It will also train women how to react when their partner becomes violent, without antagonizing him.
The *seventh module* will focus on support and solidarity. Participants will consider the value of women’s solidarity; learn how to prepare personal safety plans, identify allies, map a support network; and pledge to support to one another.
In the *eighth module*, participants will examine their changed beliefs, discuss how they should act on such changes, plan change in greater detail, envision their life without violence and the changes that this will require, and develop an action plan.

**Table 2:** Summary of intervention activities to reduce violence and increase condom use among FSWs and their intimate partners

Target group	Activity type	Activity summary
Female sex workers	Participatory reflection sessions	Workshops will bring together FSWs from the same village, meeting 10–12 times for 2 h, to complete an 8 module curriculum plus structured reflection process. Sessions will be led by a female facilitator.
	Development of safety plans	Facilitators will help each FSW develop her own safety plan.
	Counselling support	One-to-one counselling will be provided to individual FSWs by facilitators on a as needed basis.
	Linkage to services	FSWs will be linked to STI and HIV testing and care services as needed. This will include provision of male and female condoms.
	Leadership building	2 FSWs from each village will be selected and trained to hone their leadership skills, to enable FSWs to champion the cause of violence against FSWs.
	Violence response through crisis management system	FSWs experiencing violence will have access to a 24 h hotline, which will respond within 24 h to provide emotional and legal support and medical care. This system currently exists to respond to violence by non-intimate partners (IPs) (such as police and clients) only. The crisis management system will be strengthened to enable effective support following intimate partner violence.
Intimate Partners	One-on-One sessions	Male facilitators will meet with IPs of FSWs on a regular basis to discuss issues of violence and condom use, and to motivate the IPs to attend the group sessions.
	Group sessions for partners	Group reflection sessions for IPs will be conducted during two one day workshops, one month apart. The curriculum consists of 5 modules. It is assumed 30–50 % of IPs will attend.
	Training of Champions	It is hoped that some IPs will change because of the intervention and 15 such men will be selected and trained further as local champion’s for violence-free relationships.
	Linkage to services	IPs will be referred to STI and HIV testing and care services as needed. Condoms will also be distributed regularly by the facilitators.
Couples	Couples events	Events will be organised for FSWs and their IPs once every 3 months. These events will celebrate loving and responsible relationships, provide a space for couples to meet other couples and enjoy time with their partners.
	Couple counselling	Counselling will be provided to couples on an as needed basis to help resolve relationship issues. FSWs have requested such couple counselling sessions to support them in addressing issues of trust, risk, violence, condom use etc with their IPs. In these sessions, facilitators will work with both partners to support them in resolving issues while promoting equity and a positive outcome for the FSW.
Sex worker Community Based Organisation (CBO)	Training of CBO members	CBO members will receive training to enable them to understand and respond to violence related to IPs. This will build on previous training which focused on responding to violence by clients, the police or street criminals.
	Strengthening of crisis management teams	Through this project the crisis management system (which currently responds to violence by clients, the police or street criminals), will be strengthened and effective support will also be provided to FSWs experiencing intimate partner violence. FSWs who experience violence will be linked with other services like medical care, legal support or other psycho-social support.
General Population (where FSWs and their IPs live)	Folk Shows and Street Plays	Folk shows and street play teams that focus on issues of IPV/DV and raise awareness about the rights and laws regarding IPV/DV will be conducted in all intervention villages twice in the intervention period.
	Community Events	Community rallies and campaigns will be conducted in association with the other CBO programs to raise awareness on issues of IPV/DV among the general community. These rallies will aim to publicly exhibit the strength and solidarity among FSWs on issues of violence.

**Table 3:** Workshop curriculum for Intimate Partners

The workshop series for men consists of five modules in which participants learn about HIV risk, women’s rights, and laws relating to violence; share their experiences; examine their attitudes, feelings, ideals of masculinity, and behaviour; and are encouraged to practice cooperation and communication rather than confrontation and coercion. The *first* module will focus on building trust and communication. The *second* module will examine participants’ gender ideals and ideas around masculinity. The *third* module will teach participants about unsafe sex and risk and how to reduce risk and behave responsibly in intimate relations. The *fourth module* will focus on understanding violence and the role men play in stopping violence. In the *fifth* module, participants will focus on changing and considering their future which is safe and responsible.	

### Evaluation question

This study aims to assess the impact of *Samvedana Plus* on FSW vulnerability to HIV and IPV. Specifically, we aim to:

1. Assess the immediate and long term impact of the intervention on consistent condom use and experience of violence in intimate relationships among FSWs who have access to the intervention.
2. Assess the impact of the intervention on consistent condom use and use of violence in intimate relationships among the partners of FSWs who have access to the intervention.
3. Explore how the intervention has affected the response to partner violence among FSWs and the general population where FSWs and their IPs live.
4. Investigate the processes and causal pathways through which change occurs in the following areas: sense of self-worth, and individual and collective efficacy; critical thinking on gender, violence, social norms and HIV risks among FSWs and their IPs; sense of safety and well-being among FSWS; appreciation among FSWs and their IPs of STI/HIV risks in the context of intimate partnerships; awareness among FSWs, their partners and other stakeholders of violence, rights and the law; and acceptance of IPV by FSWs.

### Evaluation design

The evaluation will employ a cluster-randomised controlled trial design, with 50 % of the village clusters (intervention villages) randomly selected to receive the IPV intervention for the first 24 months and the remaining 50 % (control villages) receiving the standard HIV prevention activities implemented in Bagalkot since 2003 under the India *Avahan* programme, and transitioned to the Government of Karnataka in 2012 [67, 69]. The control villages will start to receive the intervention at month 25. The evaluation design will involve quantitative and qualitative assessments with all FSWs who report an IP; quantitative and qualitative assessments with IPs; and process/ implementation monitoring. The study will compare outcomes in intervention and control villages with FSWs at midline (12 months) and endline (24 months), and with IPs at endline (24 months). The primary analysis will compare intervention and control communities at follow-up using data from the quantitative surveys. Qualitative case studies will be done to investigate the processes and pathways through which positive change does or does not occur in terms of partner relations, violence, and negotiation of safer sex. Factors and situations that assist or prevent FSWs from using new skills related to preventing IPV and increasing condom use will be explored. Ten couples, where both members of the pair agree to be interviewed (separately) and an additional 5 FSWs and 5 IPs where only one member of the pair will be interviewed will be purposively selected and followed forward in time. These individuals will be interviewed three times over the study period. In-depth interviews with facilitators will explore how they perceive changes in relationship dynamics among FSWs and their IPs, the challenges they face, and how they themselves have internalized programme messages on the unacceptability of partner violence.

### Site selection and randomization

Bagalkot district in northern Karnataka was purposively selected from the 30 districts in Karnataka, due to high rates of HIV among both the general population (3.5 % ante-natal clinic attendees; unpublished data, NACO Antenatal surveillance, 2010) and FSW population (17.6 %; unpublished data, NACO High Risk Group surveillance, 2012). The district is comprised of six taluks, or sub-districts, of which two (Jamkhandi and Mudhol) were purposively selected for the study site due to the high numbers of FSWs estimated to be living and working in these taluks, and the large proportion of FSWs belonging to the *devadasi* community (Unpublished data, KHPT, FSW site validation, 2010). Mapping and enumeration was conducted for all FSWs living in all villages (*n* = 92) and towns (*n* = 5) of these two taluks to identify FSWs (i) who have one or more IP(s); or (ii) who frequently change their IP(s); or (iii) whose IP has more than 1 sexual partner. 74/92 villages and 5/5 towns had at least 10 FSWs with any of the above 3 characteristics. An intensive IPV intervention involving group counselling sessions was initiated in 27 villages and all 5 towns in 2011, leaving 47 villages without an intervention. These villages have an estimated 800 FSWs with an IP, and all 47 villages were enrolled in the study.

**Fig. 1:**
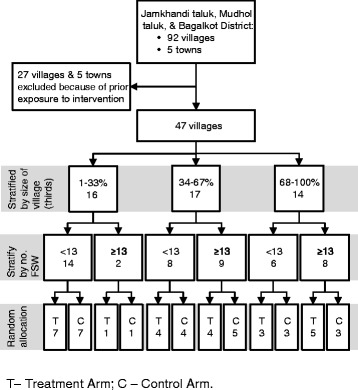
Consort Diagram for *Samvedana Plus* Trial

The village is the unit of randomization. In order to allocate villages into two cohorts, we stratified villages using two criteria: i) village population size; and ii) the number of FSWs with IPs (above or below 12). Three strata of village population size (1–33 percentile; 34–67 percentile; 68–100 percentile) and two strata of the number of FSWs with IPs (<=12 FSWs and >12 FSWs) were created, giving a total of 6 strata. Randomization of villages was then done within each strata using STATA, with half the villages (*n* = 24) randomized into the intervention arm and the remaining half (*n* = 23) randomised into the wait-list control arm (Fig. 1).

### Recruitment and inclusion criteria

The evaluation will include approximately 800 FSWs with IPs living in 47 villages (average 17 women per village). A list of FSWs and their IP status is held and regularly updated by the CBO; this will be used to identify FSWs for the quantitative surveys. The study will enroll all women in the 47 villages who engage in commercial sex work, are aged over 18, and either have an IP or have had an IP within the last 6 months. The study will also seek to enroll the IPs of these FSWs, who may or may not live in the same village as the FSW.

### Study outcomes

The study is designed to assess two primary outcomes using data from the quantitative surveys with FSWs:

(i) the proportion of FSWs who report experiencing physical or sexual partner violence in the past 6 months; and
(ii) the proportion of FSWs who report consistent condom use in their intimate relationship, within the past 6 months.

Secondary outcomes will assess the impact of the intervention on the pathways hypothesized in the Theory of Change model as potentially affecting IPV and condom use levels, including the proportion of FSWs who report:

(i) Increased self-efficacy to negotiate condom use and HIV/STI testing by their IP;
(ii) Reduced acceptance of violence by their IPs;
(iii) Increased disclosure of IPV;
(iv) Increased solidarity among FSWs around issues of IPV;
(v) Increased knowledge of self-protection strategies and sources of support for IPV.

Additional analyses will investigate the impact of IPV on the mental health of FSWs, and seek to understand how violence and mental health interact to enhance HIV vulnerability. We have not included data from IPs in the primary and secondary trial outcomes as it is uncertain how many IPs will agree to participate in either the intervention or quantitative surveys, and thus it is not possible to power the study based on anticipated IP uptake or response rates. The study will aim to assess the proportion of IPs who report (i) perpetrating violence; and (ii) consistent condom use in the past 6 months with their FSW, as well as the secondary outcomes detailed above for FSWs. Data on the fidelity and exposure to the intervention will be collected from operational records and logs.

### Quantitative data collection and data management

The project indicators will be measured with FSWs at baseline, mid-line (12 months) and end-line (24 months), and with their IPs at baseline and end-line. Cross sectional surveys will be conducted in both intervention and control arms using a face-to-face interviewer-administered questionnaire. Interviewer gender will match that of participants. Each study participant will be assigned a unique identifier, enabling questionnaires from each time-point to be linked. The surveys will include questions on primary and secondary outcomes, as well as programme exposure variables. For each of the secondary outcomes, broad indicator domains have been delineated and questions have been developed for each of the subject domains. The quantitative survey data will be double-entered and cleaned using CSPro. Statisticians assessing the outcomes will be blinded to group assignment.

### Power calculations

An initial assessment of FSWs with IPs estimated an IPV prevalence (past 12 months) of 47 %, and a consistent condom use prevalence (past 12 months) of 38 %. We calculated the power of the study to detect a difference in the prevalence of IPV between trial arms, with 800 FSWs, assuming a 10 % refusal rate. Table 4 shows the power calculations based on an IPV risk of 47 %, with varying effect sizes and risk ratios, and at different “k” values (coefficient of between-cluster variation). The power calculation was performed by analysing simulated data from 800 women, distributed across clusters using empirical data from the initial assessment. The analysis was a t-test of the cluster-level proportions of women having experienced IPV in the last year between intervention and control arms. The power calculation did not account for the stratification that was subsequently used in the randomisation, which will likely increase the power by reducing the mean within-strata value of *k* while only minimally reducing the degrees of freedom. The calculations also did not include adjustment for baseline levels of the outcome, as the correlation over time was not known. Including this in the final analysis will increase the precision of the estimate. The simulations were performed 1500 times, with a control proportion of 47 %, for different levels of *k*, an alpha of 0.05, and a narrow range of feasible effect sizes. Results suggest that the trial will have >80 % power to detect a risk ratio of 0.77, if k is between 0.15 and 0.25. This risk ratio corresponds to a risk difference of 11 %, or 44 women who be experiencing IPV were it not for the intervention.

**Table 4:** Power to detect a difference in IPV exposure (past 12 months) between study arms

Risk	K	RR	% reduction	Power (95 % CI)
47 %	0.15	0.75	25 %	88 %
47 %	0.15	0.77	23 %	84 %
47 %	0.15	0.8	20 %	73 %
47 %	0.2	0.75	25 %	86 %
47 %	0.2	0.77	23 %	82 %
47 %	0.2	0.8	20 %	70 %
47 %	0.25	0.75	25 %	89 %
47 %	0.25	0.77	23 %	85 %
47 %	0.25	0.8	20 %	70 %

Results of the power calculations is based on 800 FSWs with IPs, distributed across 47 clusters according to empirical data from the study site. The power is presented for a range of values of *k* and potential effect sizes expressed as risk ratios and percentage reductions in the risk.

### Comparing outcomes between arms

The primary outcome will be an adjusted, cluster-level intention to treat analysis, comparing outcomes in intervention and control villages at mid-line and follow-up among FSWs and at follow-up among IPs. We will perform appropriate analyses for stratified trials on the cluster mean-summaries of the primary outcomes, using the t-statistic to assess the differences observed between the arms. Adjustment for baseline measures of the outcomes, and also for variables that appear to be imbalanced at baseline, will be conducted using the ‘two step method’ that is recommended in Hayes and Moulton *Cluster Randomised Trials.* While this analysis will give a valid estimate of effect, and is robust, it may not be statistically optimal given the variation in the size of the clusters. Therefore, we will also perform an individual analysis with a mixed effects logistic regression with random effects for the village to account for clustering as well as dummy-variables for the strata, and individual-level and cluster-level potential confounders. The results of both of these analyses will be reported, and any discrepancies will be explored. Stata 14 will be used for all analyses.

Similar methods will be used to compare secondary outcomes, key indicators, and short- and medium-term outcomes. Data will be mapped onto the Theory of Change and used to interrogate the theory. Data on implementation fidelity will be used to help understand magnitudes and variability of effects of the intervention.

### Availability of data and materials

Two to three years after trial completion, endline quantitative data sets (on which the conclusions of the Trial will be based), will be deposited in a publicly available repository, R4D.

### Ethical considerations

Conducting a study around IPV and condom use requires careful consideration of the potential benefits and harms that may be caused for those involved in the research. Extensive discussions between members of the CBO, KHPT programme implementers and LSHTM were held to identify strategies to protect the safety of participants and researchers, and to try to minimize any social harms resulting from the research. In the context of partner violence research, confidentiality is both a foundation for respecting participant privacy and a strategy for limiting harmful fall out that may occur if others deduce the nature of the research. Therefore training and strict guidelines will be used with the field team to emphasize the importance of confidentiality as a cornerstone of the research. The identity of participants and the information shared by them will not be revealed to anyone who does not work on the research study. In addition, unique identifying numbers will be used to identify the questionnaires; no identifying names will be entered with the computer data. To limit stigma and possible retaliation from abusive partners, the study will be referred to in the community and with other family members, as a study on women and men’s relationships. Respondents will be informed of the true nature of the study as part of the informed consent process. We will assume that women know best how to limit their own risk. FSWs and their IPs will be interviewed separately. Discussions with CBO members running the project note that FSWs usually live independently and have their own source of income, making them less vulnerable to threats of retaliation than other women might be.

The study has obtained appropriate ethical clearances by the St. John’s Medical College and Hospital Institutional Ethics Committee, Bangalore, India and by the Observational/ Intervention Research Ethics Committee at the London School of Hygiene and Tropical Medicine, London, UK. A broad-based Community Advisory Board (CAB) has been established in Bagalkot district and will meet quarterly to review and advise on the evaluation progress as well as to monitor that the protocols are followed. Interviews will be conducted in a sensitive and non-judgmental manner, in private. The location for the interviews will be determined based on the comfort of the respondent. Appropriate procedures will be followed to obtain respondents’ witnessed, informed consent. During consent, access telephone numbers to crisis response teams will be given to women. The interviews will be one-on-one, and there will be a code to stop talking if anyone interrupts, and a plan to start the interview over at a new time or place will be decided prior to the interview if this occurs. The main potential area of distress for FSWs relates to disclosing any violence or coercion by their IP. To address this issue, the study will take great care to minimize the potential for distress or harm—including careful wording of questions to ensure that they are non-judgmental, training interviewers on how to respond if someone discloses violence or requests assistance, and providing participants with information about local sources of support.

## Discussion

*Samvedana Plus* is, to our knowledge, the first cluster randomised controlled trial (RCT) to assess the impact of a multi-level intervention on IPV and consistent condom use within the intimate partnerships of FSWs in India, and one of the first globally. It uses a rigorous methodology designed to minimise (unmeasured) confounding and several forms of selection and measurement bias.

The cluster randomised design will reduce problems associated with confounding factors, and there is sufficient geographic dispersion of the villages to minimize spill-over effects. While randomization is conducted to ensure that the arms of cluster RCTs are not systematically imbalanced, it is possible that imbalances will arise due to chance. We expect that the arms of this trial will be suitably balanced for the following reasons. First, this trial has a relatively large number of clusters and individual respondents. Second, the villages included in the design have similar characteristics in terms of sex work and relationships between sex workers and their IPs, as well as the norms that govern such relationships. Third, we stratified the randomization by the number of FSWs with IPs in the village, and then by village size. The similarity of the intervention and control samples will be assessed at baseline, and any differences adjusted for in the final analysis.

We aim to keep selection bias to a minimum through the randomization of the study villages and the selection of *all* FSWs with a current or recent IP living in the study villages. The effects that are observed, and any variability, will be explored using data on the fidelity and intensity of delivery in different sites, and the data on the context of intervention delivery. Selection bias will be largely addressed as a result of the careful process of randomization and selection of subjects. Selective loss to follow-up could also lead to bias, for example, if women who are experiencing IPV are more likely to drop out of the study. We plan to minimize this effect in the intervention villages, through the longstanding relationship that the implementation teams have established with sex workers and the trust created through the informed consent process. In addition, we expect some loss to follow-up of participants, for example due to migration for sex work or other reasons. We have allowed for 10 % loss to follow-up in our sample size calculations. We plan to reach women who have migrated away by networking with the CBO in the area to which they have migrated, if it is known. It will not be possible to mask the interviewers to the study arms; interviewer bias will be addressed by rigorous training of the field investigators. The study may also be subject to reporting bias, especially with an issue as sensitive as IPV and condom use within intimate partnerships. We will be assessing the main study outcomes through face-to-face interviews, primarily as most FSWs in this region are illiterate, and could not self-complete a questionnaire. We plan to minimise reporting bias by drawing upon the long-standing experience the research team has in conducting research on violence. The violence exposure questions used in the questionnaire have been validated [16] and will be preceded by an introduction, framing domestic violence as something that many people experience in their lives, reassuring participants on confidentiality, and explaining the importance to the study of answering questions honestly. Previous experience suggests that recruiting IPs for the study may be a challenge, and we recognize that it may not be feasible to reach a sample size large enough to measure statistically significant changes among IPs. To increase the response rate among IPs, men will be recruited from a line-list of IPs, and initially contacted by peer educators from the CBO, rather than directly by the research team. In any case, changes in condom use and violence as reported by IPs are not considered as primary outcomes, but will still provide useful information. Finally, due to the implementation of the *Avahan* Intervention programme in the district since 2004, which included a violence prevention component aimed at addressing violence by clients, police and others [29, 67], the control villages are not pure controls; this may mean the level of effect is smaller than might otherwise have been the case.

In conclusion, despite increasing efforts to reduce IPV among women in the general population, and to address ‘workplace’ violence by clients, police, pimps and others among FSWs, this is the first RCT of which we are aware to evaluate the impact of an intervention which aims to reduce domestic violence and increase condom use in the context of intimate partnerships of FSWs. Addressing the violence experience of FSWs will be key in ensuring that HIV prevention efforts are successful. This trial will provide important findings that can be used to inform programme design and policy, both in the HIV and violence prevention fields. The trial will also provide evidence on the feasibility of the approach of addressing IPV among FSWs in this setting.

## Additional file

Additional file 1: Theory of Change conceptual model for *Samvedana Plus*. (PDF 56 kb)
