# Supplementary figures and images for: Reducing violence and increasing condom use in the intimate partnerships of female sex workers: study protocol for Samvedana Plus, a cluster randomised controlled trial in Karnataka state, south India
Source: BMC Public Health. 2016 Jul 29;16:660. doi: 10.1186/s12889-016-3356-7 (PMC4966746; doi:10.1186/s12889-016-3356-7)

# Intervention with FSWs with NPIPs - program – Theory of change

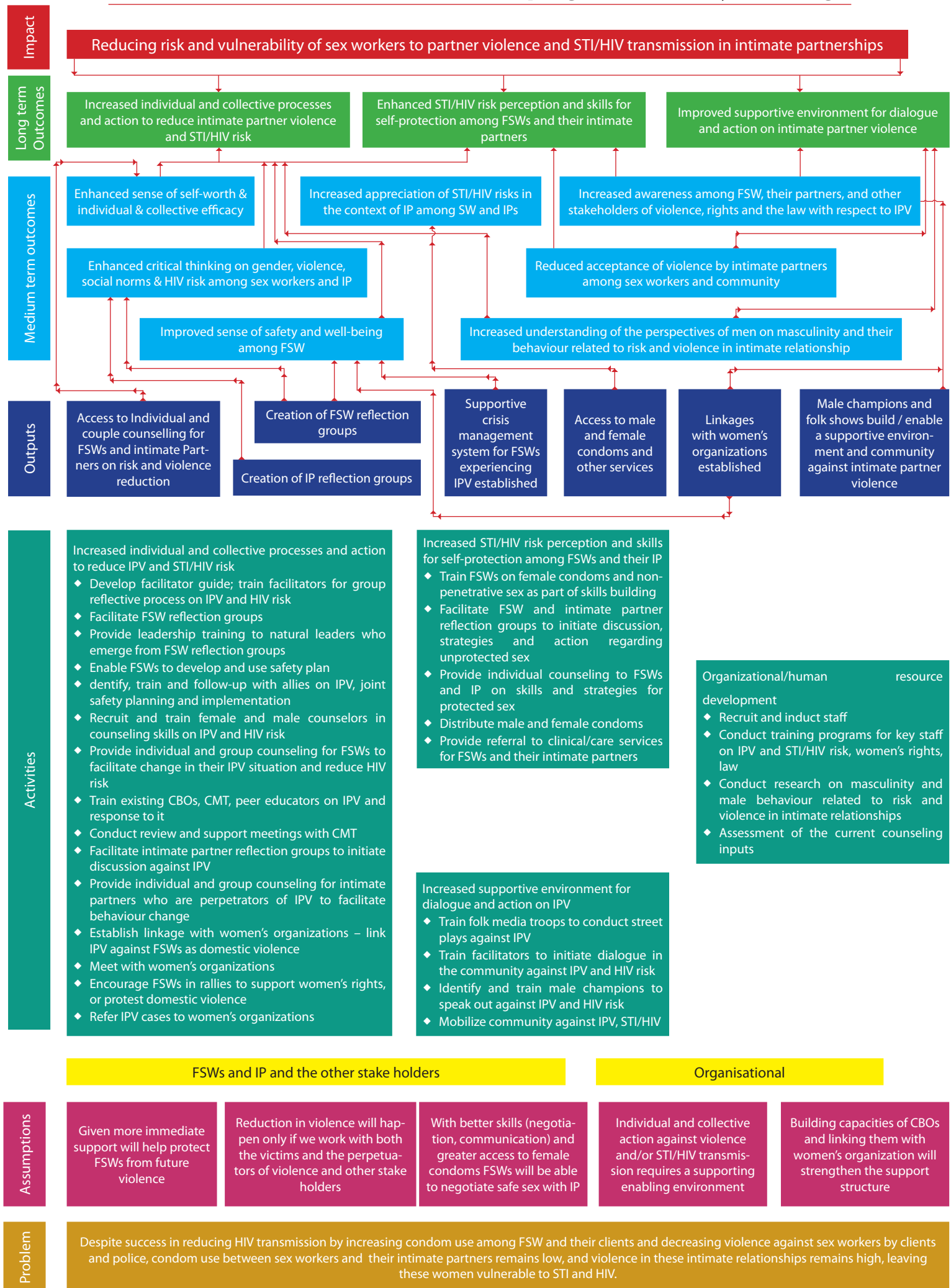

Supplement: Additional file 1: — Theory of Change conceptual model for Samvedana Plus. (PDF 56 kb) [file 12889_2016_3356_MOESM1_ESM.pdf]
